# Soil resistance and recovery during neotropical forest succession

**DOI:** 10.1098/rstb.2021.0074

**Published:** 2023-01-02

**Authors:** Masha T. van der Sande, Jennifer S. Powers, Thom W. Kuyper, Natalia Norden, Beatriz Salgado-Negret, Jarcilene Silva de Almeida, Frans Bongers, Diego Delgado, Daisy H. Dent, Géraldine Derroire, Mario Marcos do Espirito Santo, Juan Manuel Dupuy, Geraldo Wilson Fernandes, Bryan Finegan, Mayra E. Gavito, José Luis Hernández-Stefanoni, Catarina C. Jakovac, Isabel L. Jones, Maria das Dores Magalhães Veloso, Jorge A. Meave, Francisco Mora, Rodrigo Muñoz, Nathalia Pérez-Cárdenas, Daniel Piotto, Esteban Álvarez-Dávila, Yasmani Caceres-Siani, Coralie Dalban-Pilon, Aurélie Dourdain, Dan V. Du, Daniel García Villalobos, Yule Roberta Ferreira Nunes, Arturo Sanchez-Azofeifa, Lourens Poorter

**Affiliations:** ^1^ Forest Ecology and Forest Management Group, Wageningen University & Research, P.O. Box 47, 6700 AA Wageningen, The Netherlands; ^2^ Soil Biology Group, Wageningen University & Research, P.O. Box 47, 6700 AA Wageningen, The Netherlands; ^3^ Department of Ecology, Evolution, & Behavior and Plant & Microbial Biology, University of Minnesota, Saint Paul, MN 55108, USA; ^4^ Programa Ciencias Básicas de la Biodiversidad, Instituto de Investigación de Recursos Biológicos Alexander von Humbold, Colombia; ^5^ Departamento de Biología, Universidad Nacional de Colombia, Bogotá, Colombia; ^6^ Departamento de Botânica, Centro de Biociências, Universidade Federal de Pernambuco, Recife, Pernambuco CEP 50670-901, Brazil; ^7^ CATIE-Centro Agronómico Tropical de Investigación y Enseñanza, Turrialba, Costa Rica; ^8^ Smithsonian Tropical Research Institute, Roosevelt Ave. 401 Balboa, Ancon, Panama; ^9^ Biological and Environmental Sciences, University of Stirling, Stirling FK9 4LA, UK; ^10^ Max Planck Institute for Animal Behaviour, Konstanz, 78315, Germany; ^11^ Department of Environmental Systems Science, ETH Zürich, 8902, Switzerland; ^12^ Cirad, UMR EcoFoG (AgroParistech, CNRS, Inrae, Université des Antilles, Université de la Guyane), Campus Agronomique, Kourou, French Guiana; ^13^ Departamento de Biologia Geral, Universidade Estadual de Montes Claros, Montes Claros-MG CEP 39401-089, Brazil; ^14^ Centro de Investigación Científica de Yucatán A.C. Unidad de Recursos Naturales, Calle 43 # 130(32 y 34, Colonia Chuburná de Hidalgo, C.P. 97205 Mérida, Yucatán, México; ^15^ Departamento de Genética, Ecologia & Evolução, ICB, Universidade Federal de Minas Gerais, 30161-901 Belo Horizonte, Minas Gerais, Brazil; ^16^ Instituto de Investigaciones en Ecosistemas y Sustentabilidad, Universidad Nacional Autónoma de México, CP 58190 Morelia, Michoacán, México; ^17^ Departamento de Ecología y Recursos Naturales, Facultad de Ciencias, Universidad Nacional Autónoma de México, Coyoacán, Mexico City CP 04510, México; ^18^ Centro de Formação em Ciências Agroflorestais, Universidade Federal do Sul da Bahia, Itabuna-BA 45613-204, Brazil; ^19^ Open and Distance National University, Bogotá, Colombia; ^20^ Wageningen, the Netherlands; ^21^ Department of Soil and Water Systems, University of Idaho, Moscow, ID 83843, USA; ^22^ Department of Earth and Atmospheric Sciences, Centre for Earth Observation Sciences (CEOS), University of Alberta, Edmonton, Alberta, Canada T6G2E3

**Keywords:** nitrogen, phosphorus, carbon, pH, bulk density, resilience

## Abstract

The recovery of soil conditions is crucial for successful ecosystem restoration and, hence, for achieving the goals of the UN Decade on Ecosystem Restoration. Here, we assess how soils resist forest conversion and agricultural land use, and how soils recover during subsequent tropical forest succession on abandoned agricultural fields. Our overarching question is how soil resistance and recovery depend on local conditions such as climate, soil type and land-use history. For 300 plots in 21 sites across the Neotropics, we used a chronosequence approach in which we sampled soils from two depths in old-growth forests, agricultural fields (i.e. crop fields and pastures), and secondary forests that differ in age (1–95 years) since abandonment. We measured six soil properties using a standardized sampling design and laboratory analyses. Soil resistance strongly depended on local conditions. Croplands and sites on high-activity clay (i.e. high fertility) show strong increases in bulk density and decreases in pH, carbon (C) and nitrogen (N) during deforestation and subsequent agricultural use. Resistance is lower in such sites probably because of a sharp decline in fine root biomass in croplands in the upper soil layers, and a decline in litter input from formerly productive old-growth forest (on high-activity clays). Soil recovery also strongly depended on local conditions. During forest succession, high-activity clays and croplands decreased most strongly in bulk density and increased in C and N, possibly because of strongly compacted soils with low C and N after cropland abandonment, and because of rapid vegetation recovery in high-activity clays leading to greater fine root growth and litter input. Furthermore, sites at low precipitation decreased in pH, whereas sites at high precipitation increased in N and decreased in C : N ratio. Extractable phosphorus (P) did not recover during succession, suggesting increased P limitation as forests age. These results indicate that no single solution exists for effective soil restoration and that local site conditions should determine the restoration strategies.

This article is part of the theme issue ‘Understanding forest landscape restoration: reinforcing scientific foundations for the UN Decade on Ecosystem Restoration’.

## 1. Introduction

**Figure 1.**
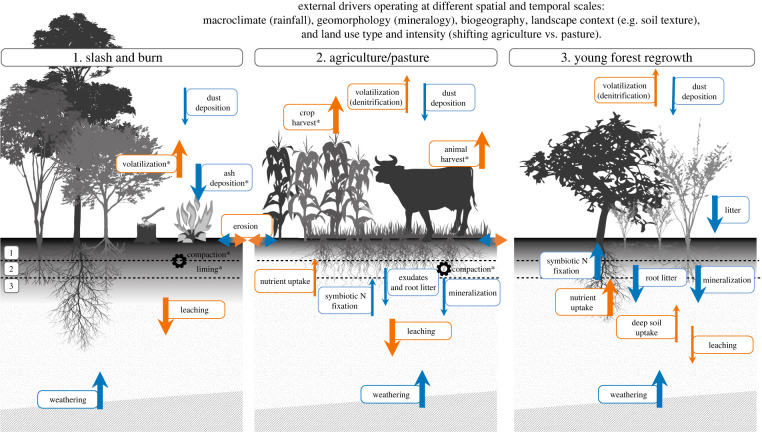
Conceptual diagram showing how nutrient (nitrogen, phosphorus) flows (arrows) change during three different phases: (1) slash and burn, (2) use as cropland (left) and pasture (right) and (3) young forest regrowth. Flows are indicated as inputs (blue arrows) and losses (orange arrows) to the soil system. Flows can be determined by different processes, e.g. decomposition [5], nitrogen fixation [6], mycorrhizal activity [7] and dust trapping [8]. Erosion can lead to nutrient input or loss, depending on the topographic position of the plot. Other processes affecting soil structure and chemistry (e.g. compaction, liming) are indicated by gears (or wheels). The magnitude of the flow is indicated by the size of the arrow. Most processes occur in all stages, and asterisks (*) indicate that the process is unique to a stage. The soil layers consist of bedrock (hatched), mineral soil (dotted) and the accumulation of organic matter in the top mineral soil layer (greyscale). Dashed lines and numbers refer to the two layers studied: (1) topsoil (0–15 cm depth) and (2) subsoil (15–30 cm depth); (3) refers to deep soil (not studied). The shifting cultivation cycle is affected by a hierarchy of external drivers (indicated on top) that operate from regional to local spatial scales, and from long to short temporal scales. Drivers included in this study are indicated in parentheses. (Online version in colour.)

Tropical forest soils are globally important for carbon and water cycling, and locally important for nutrient cycling and retention [1]. Land-use change such as deforestation for cropland or pasture is common in tropical areas. The extent to which land-use changes affect physical, chemical and biological soil properties and processes is the soil's resistance to land-use change [2–4]. Often, agricultural lands are abandoned after some years due to soil degradation and/or dominance of weedy species, after which the soils and vegetation are left to recover (figure 1). Recovering secondary forests account for at least 28% of total Neotropical forest area [9]. The resistance and recovery of tropical soils to land-use change are important locally for nutrient availability to plants and improving the water balance [10], and globally for storing large amounts of carbon [3] and cycling water [11]. Hence, for achieving the goals set by the United Nations Decade on Ecosystem Restoration (https://www.decadeonrestoration.org/), the recovery of soil conditions to support ecosystem restoration is crucial. Although we increasingly understand the recovery of above-ground forest properties following land abandonment [12,13], we know much less about the change in soil properties due to land-use change (i.e. the soil resistance) and the subsequent recovery of soil properties after land abandonment [3]. Understanding the resistance and recovery of soil properties is crucial because of the importance of soil for the recovery of both above- and below-ground biodiversity and carbon stocks, and for improving restoration practices. Here, for 21 sites spanning the Neotropics, we assess the resistance and recovery of soil physical and chemical properties in old-growth forest, during land-use for croplands and pastures and during subsequent forest succession on abandoned croplands and pastures.

Most previous studies have found that soils of regrowing forests can recover quite rapidly over time [3,14,15]. Generally, soil properties such as total organic carbon and nitrogen increase over time, and soil compaction and pH decrease over time, while evidence for plant-available phosphorus is equivocal (see below, and [5–8,16,17]). Changes in these soil properties may be caused by processes such as decomposition of litter and detrital inputs [5], symbiotic nitrogen fixation [6], mycorrhizal activity [7], nutrient uptake from deep soil layers and trapping of dust on leaf surfaces [8] (figure 1). However, the rate of recovery varies strongly among sites depending on their soil type [18], environmental conditions (e.g. climate) and land-use history [19] (figure 1). For example, high-activity clay soils (i.e. high capacity to exchange cations, and hence more fertile) and soils with high clay concentration generally have faster recovery of soil nutrients, probably because of faster vegetation regrowth [3,18]. The type and intensity of land-use before abandonment affects soil nutrients such as phosphorus [19,20]. For example, soil phosphorus may not recover if the site experienced frequent and intense burning during land conversion and pasture use [14,21]. Such changes in soil properties are generally fastest in the upper soil layer, where most decomposition of root and leaf litter takes place [3]. Many studies have assessed local-scale soil recovery (as summarized in [21]), but it remains a challenge to understand soil recovery and its geographical variation across broad-scale environmental gradients. Such generalizations are needed to underpin land-use planning and policies.

Our ability to make generalizations about how soil properties change during succession across broad geographical scales has been hampered by the availability of suitable data collected using common methods [3,22–24] rather than by knowledge gaps in our conceptual understanding (figure 1). Some studies have attempted to synthesize the broad-scale patterns and mechanisms of how secondary succession affects soil processes and properties using meta-analyses [19,23]. However, unlike forest inventories that have relatively standard measurement methods and protocols, soils can be sampled and characterized in a bewildering number of different ways. For example, studies can differ in the number of samples per plot, how samples are pooled, sampling depths and the laboratory methods used to quantify properties such as labile, available or extractable nutrients. Soil carbon inventories (e.g. absolute amount of carbon per square unit of ground area) depend on soil carbon concentration and bulk density (i.e. dry mass of soil per unit volume), both of which may be altered by land-use change [25]. Failure to account for changes in bulk density thus results in erroneous estimates of carbon loss or gain with land-use change [23,25]. These differences in methods across studies make it difficult to perform large-scale analyses for multiple soil properties.

Here, we present the first broad-scale assessment of changes in soil properties during land conversion to pasture and cropland (together referred to as ‘agriculture’), and during secondary tropical forest succession after land abandonment, using a standardized approach for field sampling and, as far as possible, for laboratory analyses. For 21 chronosequence sites comprising 300 plots across the Neotropics, we analysed six soil physical and chemical properties that are important for ecosystem functioning and nutrient, carbon and water cycling: pH, bulk density, total organic carbon (C), total nitrogen (N) and available phosphorus (P) concentrations and the C : N ratio.

We used this unprecedented dataset to ask two fundamental questions related to the resistance and recovery of soil properties. First, how do soil properties change during land conversion and agricultural use (i.e. their ‘*resistance*’, measured as the difference between soils from old-growth forests and agricultural areas), and how do such changes depend on (a) abiotic conditions (rainfall, soil mineralogy (i.e. low- versus high-activity clays) and soil texture), (b) previous land-use type and (c) soil depth? We predicted that soil carbon and nutrients will be lower in agriculture (pasture or cropland) compared to old-growth forests, probably because of volatilization during slash and burn activities, carbon and nutrient export in crops and hence lower litter inputs, and increased soil disturbance, erosion and leaching. Furthermore, bulk density and pH are expected to be higher in agricultural areas than in old-growth forest due to soil compaction by cattle or machinery, while the input of ash and reduced decomposition drive higher pH. Such changes may be strongest in the upper soil layer that may have experienced more severe depletion than deeper soils during agricultural use and where detrital inputs are highest, and in wet sites where higher productivity may lead to faster depletion of nutrients and higher rainfall to more leaching.

Second, how do soil properties recover during subsequent forest succession, and how does this *recovery* depend on (a) abiotic conditions, (b) previous land-use type and (c) soil depth? We expected that soil C and N will recover over time due to symbiotic nitrogen fixation and litter input, but can also decrease over time due to nutrient uptake by the regrowing vegetation [26]. Soil P recovery, however, depends on longer-term processes such as weathering and dust deposition (figure 1) and may therefore take longer. Furthermore, we hypothesized that (a) wetter sites may have faster recovery of soil properties because of higher vegetation productivity, root growth and litter input, but drier sites may have more rapid N accumulation because of a higher abundance of N_2_-fixing tree species [27], (b) soil recovery may be faster on abandoned crop fields than on pastures, as they are often used for a shorter period and may have been fertilized and (c) soil properties may recover faster in the upper soil layer compared to the deeper soil layer, as the upper soil layer has more fine root growth and litter decomposition. We first address these two fundamental questions, then calculate how soil budgets of carbon, nitrogen and available phosphorus change during succession to better assess the importance of different mechanisms that lead to recovery in these soil properties, and conclude with recommendations for restoration.

## 2. Methods

### (a) Site selection

**Figure 2.**
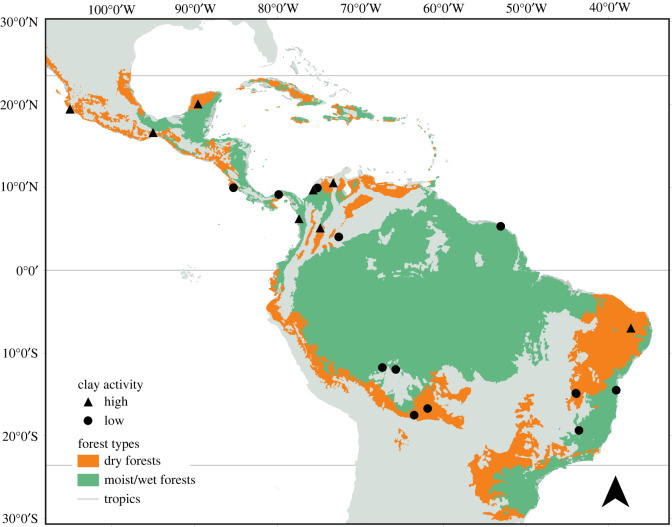
Map showing the locations of the 21 chronosequence sites. The orange background layer shows dry tropical forest area and the green background layer shows moist or wet tropical forest area. The symbols refer to the clay activity type: circles for low-activity clays and triangles for high-activity clays. (Online version in colour.)

To provide a general picture of how soil properties change during secondary succession, we collected soil samples from 21 secondary forest chronosequences across the Neotropics (figure 2). To provide a long-term perspective on how soil properties change during succession, we used a chronosequence approach by sampling areas still under active agriculture, regenerating forests of different age post-abandonment, and old-growth forests. Chronosequences use a space-for-time-substitution and assume that plots within a chronosequence are representative of the same vegetation and soil type and that most of the variation in soil and vegetation properties is therefore determined by stand age. Part of the spatial variation among plots, however, will inevitably be explained by fine-scale heterogeneity in environmental conditions (e.g. soils). Nevertheless, longitudinal studies (i.e. assessing temporal data) assessing soil recovery are rare, and chronosequence studies, therefore, provide the best opportunity to assess long-term recovery of soil properties [28], in this case, up to 95 years. Each chronosequence comprised five to 33 individual plots (300 plots in total). To evaluate whether soil properties change more rapidly in the upper soil layer compared to deeper soil layers (because of more biological activity and litter input), soils were sampled at two standardized depths (0–15 and 15–30 cm).

To evaluate how variation in soil recovery is driven by abiotic factors that vary at the regional scale (rainfall, mineralogy) and local scale (previous land-use type, clay concentration), we sampled sites that ranged widely in annual precipitation (between 750 and 3040 mm) and average clay concentration (between 4.2 and 84.8%) (figure 1). Thirteen sites had low-activity clay soils (characterized by pH-dependent charge, lower pH and cation exchange capacity and generally higher weathering), and eight sites had high-activity clay soils (characterized by permanent negative charge, higher pH and cation exchange capacity and generally lower weathering, see [3]). Nine sites were previously used for croplands, and 12 sites for pasture. One site (Arbocel in French Guiana) was clear-cut and burned but was not used for agriculture. We included this site in our analysis as a cropland site because it was one site only, and the ecological impacts would be most similar to one-time slash-and-burn cropland.

### (b) Soil sampling

For the 21 chronosequence sites, we sampled soils from active cropland or pasture (if possible), secondary forests that differ in age, and old-growth forest (see electronic supplementary material, appendix S1 for sample size and age ranges per site). Old-growth forests were defined as forests without a record of major human disturbances and were at least 100 years old.

All data were collected between 2018 and 2020. We avoided sampling after very heavy rains to avoid the influence that precipitation may have on nutrient availability. To account for spatial heterogeneity in soil properties, three soil samples were taken per sample plot, on three positions along a transect, each 5 m apart. To assess whether soil layers differ in recovery rate, we sampled mineral soil at two fixed depths: the 0–15 cm mineral soils and at 15–30 cm. In tropical rain forests, these depths include the bulk of fine root biomass [29] and are expected to be the most responsive to land-use change [3]. All chronosequence sites had a thin litter and humus layer, which was removed before sampling the mineral soil. In cases where the soil was too shallow to take a sample at 15–30 cm, only the upper soil layer was sampled. The soil from the three positions from 0–15 cm were pooled, and the same was done for the three samples from 15–30 cm, thus providing two pooled samples per plot. In total, we had 561 pooled soil samples, taken from 300 plots of different forest ages across the Neotropics.

Adjacent to the soil sampling positions for chemical analyses, soil samples were taken to determine bulk density at both depths. Bulk density is an indicator of soil compaction, and high soil compaction diminishes root growth, water storage and infiltration and increases erosion due to run-off. Furthermore, bulk density is important to convert mass-based nutrient concentrations to volume-based nutrient amounts [23,25]. To obtain bulk density, soil was sampled using a known volume, and dry mass was measured after oven-drying at 105°C for 2–5 days (until they reached constant weight). Bulk density was then determined by dividing the oven-dry mass by the fresh volume. The three bulk density values per plot per depth were averaged to obtain two values per plot, as for the other soil properties. For 77 of 561 samples, we lacked data on bulk density. To avoid exclusion of these samples for nutrient amounts and the calculated nutrient pools, we estimated bulk density values in five ways using different published formulas based on soil C and particle size distribution [30]. We predicted bulk density for the samples with known bulk density and selected the prediction that gave highest *R*^2^ values between predicted and observed bulk density (electronic supplementary material, appendix S2). Predicted bulk density values were used for the samples with missing bulk density data to calculate nutrient pools in those samples, but were not used for the statistical analyses of bulk density.

### (c) Soil chemical and physical analyses

The two pooled soil samples per plot were air-dried and shipped to four different laboratories for analyses, because of logistic or legislative limitations that prevented us from shipping them all to the same laboratory. The samples from the sites in Bolivia, Costa Rica, French Guyana, Mexico and two of the sites from Colombia (San Juan and Tolima) were shipped to the University of Minnesota. All samples from Brazil were shipped to Embrapa Amazônia Ocidental in Manaus, the samples from Panama to Smithsonian Tropical Research Institute in Panama, and samples from the four other Colombian sites to Doctor Calderón Labs (http://www.drcalderonlabs.com/), Bogota DC, Colombia. Across the four laboratories used for soil analyses, we used standardized methods to quantify soil physical and chemical variables (described in detail by [31]). All analyses were performed on soil fractions ≤ 2 mm. In brief, we measured pH in water using a 1 : 2.5 soil-to-solution ratio and a pH meter. Total soil organic C and N were measured on finely ground subsamples using a Costech Elemental Analyzer (electronic supplementary material, appendix S3). Particle size distribution was measured with a Malvern Mastersizer 3000 [32] after pretreatment overnight in 0.5% sodium hexametaphosphate and 0.5% sodium hypochlorite. Extractable soil P was determined using Mehlich 3 solution and PO_4_ concentrations were quantified colorimetrically using the ascorbic acid protocol [33]. Mehlich 3 P is thought to represent a labile or plant-available pool and has been measured widely across the tropics [34]. For some of the analyses, there were small differences in the methods used between laboratories, see electronic supplementary material, appendix S3.

### (d) Soil response variables

To assess changes in soil conditions, we used six soil properties: pH, bulk density, total organic carbon (C), total nitrogen (N), extractable phosphorus (P) and the ratio between C : N. This ratio reflects multiple processes, such as the nitrogen concentration of the inputs and the extent to which litter is transformed to humus, which leads to declining soil C : N ratios over time. pH is important for the availability of essential nutrients, especially P, and the availability and hence toxicity of aluminium. Bulk density is important for water infiltration and soil workability for agricultural use. Soil C, N and P pools are important for plant nutrient availability, and C is additionally important for below-ground carbon storage. Organic C also enhances soil nutrient and water adsorption, soil structure and biodiversity [35]. We expressed C, N and P on a volume-basis by multiplying the mass-based concentration by the bulk density. We used volumetric concentrations (i.e. the total or plant-available (for P) pools) to indicate the total nutrient availability per unit soil area, which is a better measure of nutrient stocks and may therefore better reflect the nutrients available to plants within the area explored by their roots. Not accounting for bulk density differences among samples and assessing nutrient concentrations instead of nutrient pools can lead to a general underestimation in results (electronic supplementary material, appendix S4) if soils decompact during secondary succession. Changes in bulk density, while sampling over constant, predefined soil depths, result in non-equivalent soil masses being compared [36].

### (e) Drivers of soil resistance and recovery

To understand how external drivers shape resistance and successional recovery of soil conditions, we used additional information on climate, clay concentration and mineralogy and land-use history. For climate, we used data on annual precipitation because this is often related to above-ground biomass stocks and recovery [12,37], and climatic water deficit because this represents the potential drought stress of the ecosystem. Precipitation was obtained from a local climatological station and climatic water deficit (in millimetres per year) from https://chave.ups-tlse.fr/pantropical_allometry.htm#CWD. For soil mineralogy, we classified sites into high-activity versus low-activity clays. Soils dominated by low-activity clays such as kaolinite and gibbsite are typically highly weathered, have low pH and base cation concentrations and variable charge. By contrast, high-activity clays have minerals such as montmorillonite, vermiculite and illite, display large surface area and higher base cation exchange capacity and have a constant negative charge [3]. To classify the sites into low or high-activity clay soils, we overlaid site coordinates onto the IRSIC (International Soil Reference and Information Centre) soil taxonomy grid and categorized sites mapped as Cambisols, Leptosols, Luvisols or Regosols as high-activity clay soils, and sites mapped as Ferrasols or Acrisols as low-activity clay soils following Veldkamp *et al*. [3]. Furthermore, we used the clay concentration of the site to describe differences in particle-size distribution, because soils with high clay concentration generally have high soil organic matter [38] and high above-ground productivity [39], which can all influence soil recovery. To assess the role of previous land-use type, we classified the sites as abandoned after use for cropland or pasture. Note that for assessing soil resistance (i.e. the difference in soil properties between old-growth forest and agricultural sites), land use refers to previous and current land use. However, for consistency, we refer to ‘previous land use’ only. To obtain more site-specific data on the land-use history, we also gathered information from the local investigators on the intensity of previous land-use and the frequency of fire (electronic supplementary material, appendix S5). Because of the low detail and high uncertainty of this information, we only used it as descriptive information of our sites and did not include it in any of the statistical analyses.

### (f) Statistical analyses

To assess how soil conditions change during succession and what factors determine these changes, we built two linear mixed models per soil property (bulk density, pH, C, N, P and C : N as dependent variables, *N* = 464): one model to assess resistance and one model to assess recovery. First, we assessed resistance based on all samples collected from recently abandoned agricultural sites and areas still in use (all with a forest stand age of 0 years) and samples from old-growth forests. These models included as fixed predictors stand age group (0 years versus old-growth), soil depth (upper 0–15 cm versus lower 15–30 cm), annual precipitation, previous land-use type (cropland versus pasture), clay activity type (low versus high), per cent clay concentration and the interaction between stand age group and the other predictors to assess how they influence the soil resistance. Furthermore, plot nested within site was included as a random intercept to correct for the nested design with multiple samples per site and the two samples (for the two depths) per plot. Second, we assessed soil recovery (i.e. the change during succession) based on all samples except old-growth forests, as we have no good age estimation for these plots. We used the same structure of fixed and random effects as for the models of resistance, but with stand age as a continuous predictor. Fixed predictor variables were not correlated (electronic supplementary material, appendix S6), and thus did not pose problems of multicollinearity.

For both models, to be able to compare how different drivers affect the response variables, we assessed standardized effect sizes by scaling all variables (by subtracting the mean and dividing by the standard deviation) prior to analyses. Phosphorus concentration data and C : N data were log_10_-transformed to obtain normally distributed residuals. Mixed models were run using the lmer function of the lme4 package [40]. To assess the significance of each predictor variable and interaction, we used the anova function with a Type-II test. To assess whether other models would be better fitted to the data, we compared these models with (1) models that additionally included a random effect of the site on the slope of stand age (thus accounting for differences in the successional change between sites), (2) models that included climatic water deficit instead of annual precipitation (as deficit in the dry season could be a more constraining factor for vegetation regrowth and soil processes than total annual rainfall) and (3) models that included log_10_-transformed values for stand age to assess a potential nonlinear effect (i.e. saturating effect) of stand age on recovery of soil properties. We included a log_10_ transformation instead of a quadratic polynomial to facilitate the incorporation of interactions between stand age and the other predictors and have fewer predictor variables in the model. In all cases, the models without random slopes had a lower Akaike information criterion (AIC), meaning that they better explained the data. The models with annual precipitation had either a lower AIC or did not differ substantially in AIC (i.e. less than 2 AIC units difference) compared to the models with climatic water deficit. The models with log_10_-transformed stand age had in most cases a higher AIC (i.e. a worse fit), and we, therefore, included a linear effect of stand age in all cases. We present only the results of these best-fitting models without random slopes, with annual precipitation, and with linear relationships. The significant interactions between stand age and the other predictors are visualized with the help of the emtrends function of the emmeans package in R, to assess the significance of the slope of stand age with soil properties at the different levels of the other predictor variables (e.g. pastures versus cropland). For visualization purposes, scatterplots of all soil properties versus stand age are shown in electronic supplementary material, appendix S7. All statistical analyses were conducted using R v. 3.6.1 [41].

## 3. Results

**Figure 3.**
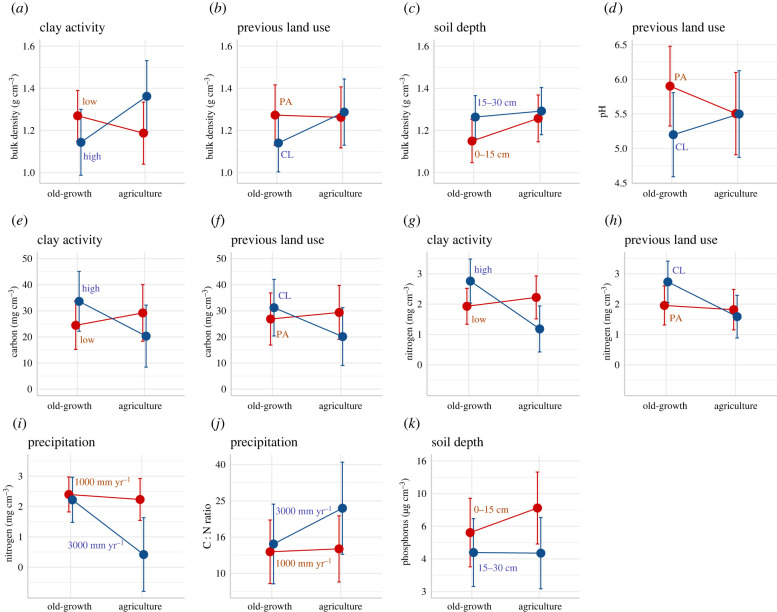
Visualization of the significant interactions between soil resistance (i.e. the differences in soil properties between old-growth forest and agricultural land) and predictor variables. For the continuous predictor variables (i.e. precipitation), the predictions are given for an arbitrarily chosen low (red) and high (blue) value. Prediction means with standard errors are shown (*N* = 174). The predictor variables are: clay activity (red = low, blue = high), previous land-use type (red = pasture (PA), blue = cropland (CL)), soil depth (red = 0–15 cm, blue = 15–30 cm), and precipitation (red = 1000 mm yr^−1^, blue = 3000 mm yr^−1^). Note that the ‘previous land use’ here refers to previous as well as current land use in the agricultural sites. Predictions are made while keeping all the other variables constant. Statistics can be found in electronic supplementary material, appendix S8. (Online version in colour.)

**Figure 4.**
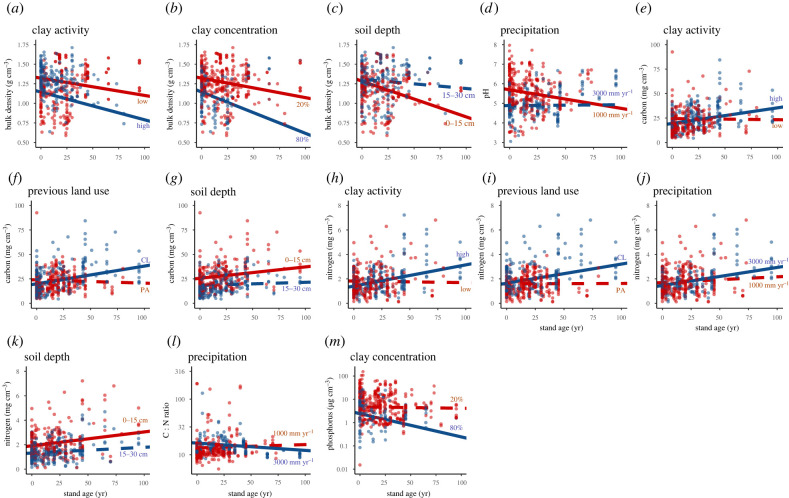
Visualization of the significant interactions between recovery (i.e. the differences in soil properties between old-growth forest and agricultural land) and predictor variables on the soil properties. For the continuous predictor variables (i.e. clay, precipitation), the predictions are given for an arbitrarily chosen low (red) and high (blue) value. Prediction means are shown (*N* = 174). The interactions between the two lines in each graph are significant. Continuous lines indicate slopes significantly different from 0, whereas dashed lines indicate slopes that are not significantly different from 0. The predictor variables are: clay activity (red = low, blue = high), previous land-use type (red = pasture (PA), blue = cropland (CL)), soil depth (red = 0–15 cm, blue = 15–30 cm), precipitation (red = 1000 mm yr^−1^, blue = 3000 mm yr^−1^) and clay concentration (red = 20%, blue = 80%). Predictions are made across the average of all other variables. The data points are coloured by level of the interaction variable. For clay concentration, red < 40% and blue > 40%, and for precipitation, red < 2000 mm yr^−1^ and blue > 2000 mm yr^−1^. Statistics can be found in electronic supplementary material, appendix S9. (Online version in colour.)

Soil properties differed between old-growth forest and agricultural lands (indicating low resistance) and changed during succession (indicating recovery), but in most cases the magnitude and direction of these changes depended on environmental conditions (annual precipitation, clay activity type, clay concentration), previous land-use type and/or soil depth (table 1, figures 3 and 4).

**Table 1.** Description of the general stand-age effect and the interactions of stand age with precipitation, clay type (low versus high activity), clay concentration (%), previous land-use type (cropland versus pasture) and soil depth (upper 0–15 cm versus lower 15–30 cm) on the resistance of soil properties (i.e. the difference between old-growth and agriculture) and the recovery of soil properties (i.e. the change with stand age). Empty cells for the interaction effects indicate non-significant effects. Note that the main effects of precipitation, clay type, clay concentration, previous land-use type and soil depth were also included in the model, but not explained here (but see electronic supplementary material, appendix S9 for statistics).

soil property	resistance / recovery	stand age	stand age × precipitation	stand age × clay type	stand age × clay conc.	stand age × previous land-use type	stand age × soil depth
bulk density	resistance (change from old-growth to agriculture)	general increase		increase at high-activity clay		increase in pastures	increase in deeper soil layer
	recovery (change during succession)	general decrease		stronger decrease in high-activity clay	stronger decrease at high clay conc.		stronger decrease in upper soil layer
pH	resistance (change from old-growth to agriculture)	decrease or no change				decreases in pastures	
	recovery (change during succession)	decrease or no change	decreases at low rainfall, no change at high rainfall				
C	resistance (change from old-growth to agriculture)	general decrease		decrease in high-activity clay, weak change in low-activity clay		decrease in croplands, weak change in pastures	
	recovery (change during succession)	general increase		increase in high-activity clay, no change in low-activity clay		increase in croplands, no change in pastures	increase in upper soil layer, no change in lower soil layer
N	resistance (change from old-growth to agriculture)	general decrease	decrease at high precipitation, no change at low precipitation	decrease in high-activity clay, weak change in low-activity clay		decrease in croplands, weak change in pastures	
	recovery (change during succession)	general increase	increase at high precipitation, no change at low precipitation	increase in high-activity clay, no change in low-activity clay		increase in croplands, no change in pasture	increase in upper soil layer, no change in lower soil layer
C : N	resistance (change from old-growth to agriculture)	increase or no change	increase at high precipitation, no change at low precipitation				
	recovery (change during succession)	decrease or no change	decrease at high precipitation, no change at low precipitation				
P	resistance (change from old-growth to agriculture)	increase or no change					increase in upper soil layer, no change in lower soil layer
	recovery (change during succession)	decrease or no change			decrease at high clay concentration, no change at low clay conc.		

### (a) Resistance

Due to land conversion and subsequent land use (as shown by the difference between old-growth and agriculture, figure 3, table 1, electronic supplementary material, appendix S8), bulk density increased at high-activity clay and cropland sites and in the upper soil layer, but did not clearly change in low-activity clays, pastures and the deeper soil layer (figure 3*a,b,c*). Due to land-use change, pH decreased in pastures and tended to increase in cropland sites (figure 3*d*). Carbon (C) and nitrogen (N) pools showed a general decrease due to land-use change, and this decrease was especially visible at high-activity clay sites and croplands (figure 3*e–h*). Nitrogen additionally decreased due to land-use change in wet sites (figure 3*i*). The C : N ratio increased due to land-use change at high precipitation but remained constant at low precipitation (figure 3*j*), and soil extractable phosphorus (P) tended to increase in the upper soil layer and remain constant in the lower soil layer (figure 3*k*).

### (b) Recovery

Bulk density generally decreased during secondary forest succession (table 1, electronic supplementary material, appendix S9). This decrease was dependent on soil depth, clay concentration and clay activity type (i.e. these variables showed a significant interaction with stand age): the bulk density decrease was especially strong in sites with high-activity clays (figure 4*a*) and high clay concentration (figure 4*b*) and in the upper soil layer and (figure 4*c*). pH decreased in sites with low annual rainfall and did not change in sites with high annual rainfall (figure 4*d*). C and N generally increased during succession, especially in high-activity clay sites (figure 4*e,h*), after cropland abandonment (figure 4*f,i*), and in the upper soil layer (figure 4*g,k*). N additionally increased during succession at high precipitation (figure 4*j*). The C : N ratio decreased during succession at high rainfall, but did not change significantly in other conditions (figure 4*l*). P decreased during succession in sites with high clay concentration but did not change in sites with low clay concentration (figure 4*m*).

## 4. Discussion

We assessed how soil properties changed from old-growth forests to agricultural use (resistance) and during subsequent forest succession (recovery), and what factors predict these changes. All soil properties showed significant changes in the resistance and recovery phases, but the direction and magnitude of change varied with environmental conditions (climate and soil), previous land-use type and/or soil depth, indicating that soil resistance and recovery are largely context-dependent. First, we will discuss the resistance and recovery of physical and chemical soil properties. Second, we will assess changes in nutrient budgets across our sites. And last, we conclude with recommendations for restoration.

### (a) Resistance and recovery of soil properties

#### (i) Bulk density

We expected that bulk density would have low resistance to land conversion and subsequent agricultural land use, and show an increase because of compaction by cattle and possibly machinery and a decrease in root density and activity of macrofauna during land conversion and agricultural use [16,42]. We found, indeed, an increase in bulk density. However, this increase was only found in high-activity clays, pastures and in the upper soil layer (figure 3*a–c*), indicating that areas with that soil type and land-use history are less resistant to land-use change. Possibly, changes are strong in high-activity clays because they are more fertile than low-activity clays and may support more fine root biomass in old-growth forest, and decomposition of fine roots during agricultural use leads to greater compaction of soils. Furthermore, pastures show an increase in bulk density because of trampling by cattle, especially affecting the upper soil layer.

Regarding recovery, we expected bulk density to decrease because of root growth by woody species [43,44], the increasing abundance, diversity and activity of macrofauna, the absence of agents that cause compaction (cattle, farm machinery), and the decline of compacting earthworms but increase of decompacting earthworms and termites [45]. As predicted, bulk density generally decreased during succession (table 1, figure 4*a–c*). This successional decrease in bulk density was stronger in the upper soil layer compared to the deeper soil layer (figure 4*c*), at high-activity clays compared to low-activity clays (figure 4*a*), and at high clay concentration compared to low clay concentration (figure 4*b*), indicating highest recovery in such areas. Decreases in bulk density are faster in the upper soil layer possibly due to higher levels of soil organic matter [46], and because woody plants mainly root in the upper soil layer where most resources are found. Veldkamp *et al.* [3] also found that bulk density recovers more quickly in the superficial soil layers. The faster decrease in bulk density at high-activity clays and high clay concentration is probably because such fertile soils lead to higher plant productivity, and therefore faster root growth, higher amounts of soil organic matter and, hence, faster decompaction.

Changes in bulk density in the deeper soil depth with forest succession (figure 4*c*) are partly caused by the decompaction of the upper soil layer. That is, if the upper 15 cm soil decompacts, then this volume increases and, in later successional stages, part of this former upper soil layer is now considered to be part of the 15–30 cm soil layer. However, as the initial differences in bulk density after forest conversion were very minor (figure 4*c*), this effect of non-equivalence of fixed soil layers was very limited in our dataset. Thus, bulk density is initially high due to agricultural land use but rapidly recovers to lower values during succession, especially in the upper soil layer and in clayey and fertile soils possibly due to more root growth, macrofaunal activity and increases in soil organic matter.

#### (ii) pH

We predicted that, during deforestation and subsequent agricultural use, soil pH would increase as a result of ash (i.e. carbonate) formation during burning. We found, however, no general difference in pH between old-growth forests and recently abandoned agricultural land, except for lower pH after abandonment in pastures (figure 3*d*), perhaps because of the accumulation of acidic compounds from incompletely decomposed grass root litter [3]. This indicates that, in most cases, pH has high resistance to land-use change.

For recovery, we expected pH to decrease during forest succession due to (1) accumulation of incompletely decomposed litter, (2) an excess of protons in the soil solution to compensate for the excess uptake of base cations by the regrowing vegetation and/or (3) leaching of base cations along with leaching of negatively charged nitrate (in cases where N inputs are larger than plant demand). As expected, we found a general decrease in pH during forest succession. This pH decrease was strong in sites with low precipitation and absent in sites with high precipitation (figure 4*d*). Dry sites have a higher proportion of N_2_-fixing tree species (at the start of succession on average 60% of the tree basal area in dry forests are nitrogen fixers, compared to 10% in moist forest, [27]). N_2_ fixation leads to plants exhibiting an excess cation uptake, and in order to maintain electroneutrality, this is compensated by exudation of protons and hence results in acidification of the soil [47]. Over time during succession, this can lead to increasing amounts of protons in the soil and continued acidification. Furthermore, in dry sites, the annual litter input may be higher because of a high abundance of deciduous tree species [48], which leads to a greater amount of partly decomposed organic material and a decrease in pH. Taken together, pH generally decreases during succession and decreases more rapidly in dry sites likely due to an increased input of partly decomposed organic material and the exudation of protons by the vegetation.

#### (iii) Carbon and nitrogen

We predicted that soil carbon (C) and nitrogen (N) pools would decrease due to land conversion and agricultural use because of volatilization during slash and burn activities, carbon and nutrient export in crops and hence lower litter inputs, and increased soil disturbance, erosion and leaching. We indeed found a general decrease in C and N due to land conversion and land-use change. This was especially strong in high-activity clays (figure 3*e,g*) probably because of a stronger drop in litter input than in low-activity clays, and was strong in croplands (figure 3*f,h*) probably because less C and N are released during decomposition from crop roots compared to the thick layers of pasture roots [49].

For C and N recovery after land abandonment, we expected that C and N would increase because of carbon and nitrogen input from root and leaf litter and because of nitrogen fixation by free-living and symbiotic bacteria (figure 1). Indeed, we found a successional increase in C and N in secondary forests on previous croplands (cf. [3,6]), high-activity clay soils and the upper soil layer (figure 4*e,f,h,i*), indicating that C and N recover toward old-growth values. N furthermore increased during succession in wet sites (figure 4*j*). Contrasting successional patterns in C and N depending on the local conditions (i.e. land-use history, soil type and soil depth) can be explained by differences in conditions at the onset of succession due to previous land-use type, and by differences during forest succession.

C and N increase during succession in former croplands but not in pastures. Possibly, the high density of grass roots in pastures is replaced by tree roots, resulting in no net change in C and N. Croplands, however, may have less dense roots systems in the upper 30 cm of the soil, and fine root growth from the recovering vegetation, therefore, leads to increases in C and N. This possibility is supported by higher initial soil C and N levels in croplands (figure 3*h*). Meta-analyses also showed that deforestation with subsequent grassland establishment increased soil organic matter (and, hence, C) storage, whereas transformation to cropland reduced soil organic carbon content ([45], but see [46,50,51]).

C and N increased during succession in high-activity clays, probably because the fertile soils support relatively faster forest regrowth [12], leading to higher litter input and, hence, faster C and N recovery. Moreover, C and N decreased during land use in high-activity clays (figure 3*e,g*, blue points), which leads to lower starting values and a potentially steeper slope. The successional increase in N in wet sites may be caused by the faster forest regrowth and higher litter input in such forests.

Due to land conversion and agricultural land-use, the soil C : N ratio increased at high precipitation (figure 3*j*) but did not change in other conditions. This increase after land conversion, and therefore higher C : N starting values, may explain the C : N decline during secondary succession at high precipitation (figure 4*m*). Furthermore, during litter decomposition, C : N ratios generally decline because organic N remains immobilized in organic matter whereas a proportion of soil carbon is released as CO_2_. This may be the case especially in wetter sites that have generally faster decomposition rates (but see [3]) and are more productive, leading to more litter input and faster changes in C : N.

Hence, C and N increase during succession in croplands, high-activity clays, wet sites and the upper soil layer probably due to high litter input from a quickly recovering forest.

#### (iv) Phosphorus

We predicted an increase in extractable P after land conversion and land-use change, due to release of P in ash after burning and lower P uptake. We found that P tended to increase in the upper soil layer, but did not change in the lower soil layer (table 1, figure 3*k*). Possibly, P did not differ strongly between old-growth forests and agricultural lands because input from burning was balanced by uptake by crops and grasses and leaching to deeper soil layers.

For P recovery, we predicted a slight decrease in extractable P during forest succession because of uptake by regrowing vegetation, and immobilization of P in organic materials. This decline would be insufficiently compensated by increasing atmospheric deposition during forest succession, as forest captures more dust than low vegetation [8], and upwards P movement from lower soil depths due to uptake and return to upper layers after litter fall. We found that P did not change in soils with a low clay concentration, and decreased during succession in soils with a high clay concentration (figure 4*m*). Soil P was significantly lower in later-successional forests (greater than 30 years) compared to old-growth forests (electronic supplementary material, appendix S10) possibly due to P uptake by the vegetation being a much faster process than P input and changes into different P forms, indicating that soil P may not or very slowly recover to old-growth values [52]. Secondary forest succession might therefore become increasingly P-limited, especially in situations where hotter fires result in larger P losses after forest conversion [53]. Soil P decreases more in clayey soils because these may have higher plant productivity and, hence, nutrient uptake.

The weak overall changes, or even decreases, in extractable P during tropical forest succession may limit the full and long-term recovery of tropical forests, especially because P is thought to strongly limit forest productivity on old, weathered and leached tropical soils [54,55]. Furthermore, it suggests a change from N-limited recovery in early succession (cf. [51]) toward P-limited recovery in late succession. Previous studies have found strong legacies of long-term agricultural use [20,56] on soils in regrown old-growth forests. Here, we show that such legacies may also exist for extractable P after slash-and-burn events followed by a relatively short use for agriculture.

### (b) Nutrient budgets

During forest recovery, soil C, N and P availability can be restored through different processes (figure 1). Tracking the inputs and outputs of elements to the soil through budgets can help identify sources of nutrients to support forest regrowth and identify gaps in our knowledge.

Carbon, although not considered a plant nutrient, is important as a source for organic N and P and for cation exchange capacity and is mainly restored when carbon input from above-ground and below-ground litter exceeds carbon losses from decomposition. Across our sites, soils in agricultural fields or in recently abandoned sites store on average 62.5 Mg C ha^−1^ in the upper 30 cm, and this soil C increases with 0.24 Mg C ha yr^−1^ (data are derived from a linear mixed model with stand age, soil depth and interaction as fixed predictors). This substantial rate of C sequestration in only the first 30 cm of the soil [57,58] is one-twelfth of the carbon sequestration rate of all above-ground vegetation during tropical forest succession [12] and is similar to the carbon sequestration rate of above-ground vegetation in old-growth tropical forests [59]. C stored in lower soil layers can also be substantial, which would further enhance total soil C sequestration [60]. This underlines the importance of soil for carbon sequestration and climate regulation.

Nitrogen is expected to be restored mainly through symbiotic N_2_-fixation by trees belonging to the Fabaceae family [61,62], which can be very abundant especially in secondary tropical dry forests [27]. Nevertheless, the abundance of Fabaceae has been found to be a poor predictor of actual N_2_-fixation and forest recovery [6,63]. Additional nitrogen sources are non-symbiotic N_2_-fixation by leaf-inhabiting cyanobacteria or lichens, non-symbiotic microbial N_2_-fixation in litter and soil layers, and release of soil organic N due to enhanced soil organic matter turnover [64,65].

Across our sites, recently abandoned agricultural lands (averaged over croplands and pastures) contain 4.37 Mg N ha^−1^ in the upper 30 cm soil, and our regression models indicate that N is sequestered at an average rate of 27.4 kg N ha yr^−1^. The gross N input is likely much larger but balanced by substantial hydrological N losses to deeper soil layers (nitrate leaching) and N losses to the atmosphere (denitrification) [66,67]. Net N accumulation and especially gross accumulation are substantially larger than the symbiotic N_2_-fixation for mature tropical forests, which has been estimated to be around 3 kg N ha yr^−1^ [68]. Secondary forests may fix more nitrogen than mature forests because of a higher proportion of nitrogen-fixing trees, high light levels that allow for high photosynthetic carbon gain and carbon supply from trees to their symbionts, and because N fixation rates are especially high when soil N levels are low [69]. For example, in early stages in secondary moist forests in Panama, symbiotic N_2_-fixation amounted to 10–29 kg N ha yr^−1^, but these values rapidly declined after 20–30 years [70].

Contrary to studies that highlight the importance of symbiotic N_2_-fixation, some studies have shown that non-symbiotic N_2_-fixation may be equally or more important for N accumulation than symbiotic N_2_-fixation [65,71]. Furthermore, substantial N input may, at least in some sites, come from natural and anthropogenic N deposition [72], and enhanced soil organic matter turnover and nitrogen mineralization in deeper soil layers can be the main source of N accumulation [64]. In sum, the high rate of N accumulation in our study cannot be explained by symbiotic N_2_-fixation alone (cf. [6,58]), but is likely the result of multiple N sources.

Extractable phosphorus can decline through plant uptake and storage in plant tissue or can increase through uptake from deeper soil layers and subsequent litter decomposition in shallow soil layers or through dust deposition. Across our sites, extractable P in (abandoned) agricultural fields was on average 28.1 kg P ha^−1^ in the upper 30 cm soil and declined during succession with an average rate of 0.17 kg P ha yr^−1^. This net decline in soil extractable P suggests that losses from the soil pool due to plant uptake exceed incoming fluxes and that the P available to plants reduces and the increasing P-limitation may hamper full forest recovery.

### (c) Implications for restoration

Most abandoned and/or degraded lands have impoverished soils [73,74]. Local farmers depend on soil recovery during the fallow period of the land for their future food production and income [75]. Efficient and effective recovery of soil quality provides the basis for large-scale ecosystem restoration (e.g. [76]) and is crucial to meet the goals of the Bonn challenge (www.bonnchallenge.org) and the UN Decade of Ecosystem Restoration (https://www.decadeonrestoration.org/). For example, the Land Degradation Neutrality of the UN Convention to Combat Desertification has defined soil organic carbon as one of their indicators to assess the quality of land resources to support ecosystem functions and services (e.g. food production) [77]. However, there is no single solution to the question of how to restore soil conditions, and best practices strongly depend on local conditions and may need complementary solutions [78]. Below we discuss the best options for soil restoration given the different local conditions that we studied.

#### (i) Potential for natural soil recovery

Decline in soil quality due to agricultural use can affect three main groups of soil processes: physical (erosion and compaction), chemical (disruption of nutrient cycles) and biological (loss of soil microbial and macrofauna diversity, abundance and activity). Erosion and compaction can be quantified from bulk density and organic matter, nutrient cycles from organic C, N and P pools, and biodiversity loss is often associated with loss of organic matter and organic carbon as this is food and habitat for soil organisms [79]. Our results highlight that most soil properties can recover naturally after abandonment of cropland or pasture. First, bulk density decreases during natural forest regeneration, thereby reducing compaction and enhancing processes such as water storage, drainage and aeration [3], and facilitating root growth, productivity and, hence, forest recovery. Second, although dependent on clay activity type and previous land-use type, on average the organic C and N pools increase during succession, which helps support a rich and productive soil system as it facilitates nutrient and water adsorption, improves soil structure, water infiltration, and soil biodiversity [35]. Third, during the first decades, soil C in the top 30 cm soil increases at a rate of about 0.24 Mg C ha yr^−1^, which is similar to above-ground carbon sequestration rate by old-growth forests [59]. Moreover, C stored in soils has generally much higher residence time than C stored in vegetation [80], increasing the soil's importance for C storage. This fast and long-term sequestration of soil organic C highlights the climate change mitigation potential of regenerating tropical forests. Secondary succession is, therefore, an inexpensive, nature-based approach to restore soils, and meet (inter)national commitments for climate change mitigation (e.g. the Paris agreement), land degradation neutrality, biodiversity conservation and sustainable development goals. Recovery of P, however, is not always guaranteed through natural recovery. If P is lost by previous land-use change, for instance through frequent and high-intensity fires, P fertilization might be necessary to foster and sustain succession.

#### (ii) Recommendations for active soil restoration

Recovery of the soil properties studied here is strongly dependent on local site conditions and is especially affected by soil clay type, clay concentration, previous land use, and precipitation. Restoration efforts should therefore be tailored to site-specific conditions. First, cropland sites and high-activity clays have naturally fast recovery of vegetation and soil nutrients (e.g. fast increases in soil C and N and decreases in bulk density), and soil recovery in these sites may not require human intervention and may recover fully through natural forest regeneration. However, pasture sites and low-activity clays have no or a slower recovery of soil properties and may need active restoration or assisted natural regeneration, such as planting of fast-growing species to restore soil carbon (and shade out competitive pasture grasses), control of aggressive competitors, and the introduction of N_2_-fixing species from the beginning of the restoration action in order to restore soil nitrogen. Second, restoration is most likely to be N-limited during early succession [81], and becomes gradually more P-limited as the forest ages. This is especially notable in sites with high clay concentration that show faster decrease in extractable soil P during succession. To facilitate restoration of P to local old-growth levels, active restoration may include the use of fertilizers or the planting of deep-rooting plants with enhanced phosphatase activity or enhanced exudation of carboxylates that are able to use P pools of lower extractability [82].

In sum, during forest succession on abandoned agricultural lands, soils recover rapidly in terms of physical properties (bulk density) and processes (e.g. decompaction, water filtration), biodiversity (supported by increasing organic C), and C and N pools, but may need assisted regeneration or restoration of soil properties, especially in sites on low-activity clays and abandoned pastures, and to counteract increasing P-limitation during forest succession. Hence, in most sites and with sufficient time and/or assisted restoration, soil properties will recover naturally and support rich below- and above-ground biodiversity and productivity. This means that, for a large proportion of abandoned agricultural lands, natural succession and forest regrowth can be used as a nature-based solution for ecosystem restoration.

## Data Availability

The data used in the analyses of this manuscript are publicly available from DANS (https://dans.knaw.nl/en). The data are provided in the electronic supplementary material [83].
